# Micro-Milling Tool Wear Monitoring via Nonlinear Cutting Force Model

**DOI:** 10.3390/mi13060943

**Published:** 2022-06-14

**Authors:** Tongshun Liu, Qian Wang, Weisu Wang

**Affiliations:** School of Mechanical and Electric Engineering, Soochow University, Suzhou 215021, China; tongshunliu@hotmail.com (T.L.); 20204229012@stu.suda.edu.cn (W.W.)

**Keywords:** micro-milling, tool wear, online monitoring, cutting force

## Abstract

Mechanistic cutting force model has the potential for monitoring micro-milling tool wear. However, the existing studies mainly consider the linear cutting force model, and they are incompetent to monitor the micro-milling tool wear which has a significant nonlinear effect on the cutting force due to the cutting-edge radius size effect. In this study, a nonlinear mechanistic cutting force model considering the comprehensive effect of cutting-edge radius and tool wear on the micro-milling force is constructed for micro-milling tool wear monitoring. A stepwise offline optimization approach is proposed to estimate the multiple parameters of the model. By minimizing the gap between the theoretical force expressed by the nonlinear model and the force measured in real-time, the tool wear condition is online monitored. Experiments show that, compared with the linear model, the nonlinear model has significantly improved cutting force prediction accuracy and tool wear monitoring accuracy.

## 1. Introduction

Micro-milling, as the name suggests, is generally milling carried out at the microscale [1]. With the high machining efficiency and the ability to cut diverse materials, micro-milling technology is widely used in the field of manufacturing ultra-precise microdevices [2]. Due to the tiny tool scale and high rotation speed, the micro-milling tool wears rapidly [3]. Tool wear not only reduces the machining quality but also increases the cutting force. Severe wear will even break the tool and damage the workpiece. To avoid the harmful effects of tool wear, it should carry out online tool wear monitoring during the micro-milling process [4].

Generally, tool wear monitoring could be categorized into two methods: data-driven monitoring and mechanism-based monitoring [5,6]. In the data-driven methods, the monitoring model is built with the tool wear data and the cutting signals such as the cutting force [7], vibration [8], and acoustic emission [9,10] collected in the practical machining process. Hsieh et al. [11] collected the vibration signals to train the backpropagation neural network for tool wear monitoring. Guo et al. [12] adopted a support vector machine to monitor tool wear conditions. To ensure monitoring reliability, the data-driven monitoring model should be learned with big tool wear data. However, as the tool in micro-milling is small, it is difficult to acquire enough micro-milling tool wear data to train the monitoring model. In mechanism-based monitoring methods, the monitoring model is built by analyzing the effect of tool wear on the cutting signals. With the prior mechanistic knowledge of tool wear, the mechanism-based method is reliable and efficient to monitor the micro-milling tool wear.

The cutting force signal is widely adopted for micro-milling tool wear monitoring due to the high sensitivity to tool wear conditions [13,14]. Recently, researchers have explored different mechanistic cutting force model-based tool wear monitoring methods. Hou et al. [15] built an analytical milling force model to monitor the flank wear width. Nouri et al. [16] and Pan et al. [17] adopted the cutting parameters-independent coefficients extracted from the mechanistic cutting force model to estimate the tool wear. Liu et al. [18] built an analytical milling force model which considers the comprehensive effect of tool wear and tool runout on the micro-milling force to estimate the tool wear under variable cutting parameters and runout.

The studies show that the mechanistic cutting force model is efficient to monitor the milling tool wear. However, the adopted cutting force models do not consider the nonlinearity caused by the cutting-edge radius-size effect, and it is incompetent to monitor the micro-milling tool wear condition which has a great nonlinear impact on the cutting force.

With the development of micro-milling, the cutting-edge radius size effect [19,20], the minimum uncut chip thickness [21,22], and the nonlinear micro-milling force caused by the size effect have been intensively investigated. Chen et al. [23] studied the nonlinear ploughing force caused by the round cutting-edge in micro-milling soft-brittle crystals. Zhou et al. [24] built a semi-analytic model to reveal the nonlinear impact of the wear-varying cutting edge on the cutting force. In our previous work, a mechanistic model with an analytic minimum uncut chip thickness is constructed to reveal the nonlinear effect of cutting-edge radius on the micro-milling force [25]. Those studies have laid a theoretical foundation for nonlinear micro-tool wear monitoring. In this study, a nonlinear mechanistic cutting force model considering the comprehensive effect of the cutting-edge radius and tool wear is constructed. A stepwise optimization method is proposed to estimate the multiple parameters of the nonlinear model. The online tool wear monitoring is carried out by minimizing the gap between the theoretical force expressed by the nonlinear model and the force measured in real-time.

This paper evolves as follows. In Section 2, the nonlinear micro-milling force model is constructed. The model parameters are estimated in Section 3. The online monitoring method is proposed in Section 4. The effectiveness of the monitoring method is experimentally validated in Section 5. 

## 2. Nonlinear Cutting Force Model of Micro-Milling

The nonlinear cutting force is mathematically represented as:

(1) $$dF_{c}=\left[\int_{0}^{h}K_{c,\mathrm{sp}}\left(h^{’}|\lambda_{s},r_{e},\alpha \right)\cdot dh^{’}+K_{c,\mathrm{vb}}\left(VB|\lambda_{v}\right)\right]dz$$

(2) $$dF_{r}=\left[\int_{0}^{h}K_{r,\mathrm{sp}}\left(h^{’}|\lambda_{s},r_{e},\alpha \right)\cdot dh^{’}+K_{r,\mathrm{vb}}\left(VB|\lambda_{v}\right)\right]dz$$

Notations $dF_{c}$ and $dF_{r}\;$ are the tangential and radial cutting forces of one cutting tooth at the unit cutting depth dz. The shear-ploughing coefficients $K_{c,\mathrm{sp}}$ and $K_{r,\mathrm{sp}}$ represent the nonlinear distribution of the shear-ploughing forces on the round cutting edge. The friction force coefficients $K_{c,\mathrm{vb}}$ and $K_{r,\mathrm{vb}}\;$ represent the relationship between the flank wear width and the friction forces in the flank wear region. A detailed discussion of the nonlinear shear-ploughing coefficients could refer to in study [25]. The friction force coefficient refers to the study [15]. Notation $r_{e}$ is the cutting-edge radius, α is the ideal rake angle, h is uncut chip thickness, VB is the flank wear width. Model parameters set $\lambda_{s}$ include the mechanical parameters in the shear-ploughing region on the round cutting edge. Model parameters set $\lambda_{v}$ includes the mechanical parameters in the flank wear region. The detailed description of the model parameters is listed in Table 1.

**Figure 1:**
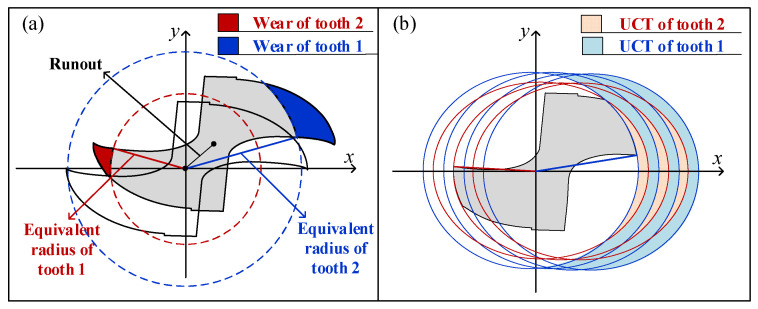
Equivalent cutting radius and uncut chip thickness. (**a**) Equivalent cutting radius under tool runout and tool wear. (**b**) Uncut chip thickness with the equivalent cutting radius.

According to study [26], the uncut chip thickness in the force model could be written as:

(3) $$h_{k}\left(\theta \right)=\max\left\{\min_{m}\left[\Delta R_{k,m}+f_{z}\sin\left(\theta_{k}\right)\frac{M\cdot \Delta \theta_{m,k}}{2\pi }\right],0\right\}$$

where $h_{k}\left(\theta \right)$ is the instantaneous uncut chip thickness of the k-th cutting tooth at the reference rotation angle θ. Notation $\theta_{k}$ is the rotation angle of the k-th cutting tooth when the reference rotation angle is θ. Notation $\Delta R_{k,m}$ is the value of the k-th equivalent radius minus the m-th equivalent radius. The difference $\Delta R_{k,m}$ is mainly affected by the tool runout and the asymmetry of multi-tooth wear. Notation $\Delta \theta_{m,k}$ is the angle by which the m-th tooth leads the k-th tooth in the direction of rotation, as Figure 1 shows. In this study, the *M* teeth are numbered from 1 to *M* along the direction of tool rotation. As the length of tool runout and the reduction of the cutting radius caused by the tool wear are much smaller than the equivalent cutting radius, the effect of tool runout and tool wear on the angle $\Delta \theta_{m,k}$ could be neglected. Therefore, the angle between the k-th original cutting radius and the m-th original cutting radius is adopted to approximately represent the angle $\Delta \theta_{m,k}$.

**Figure 2:**
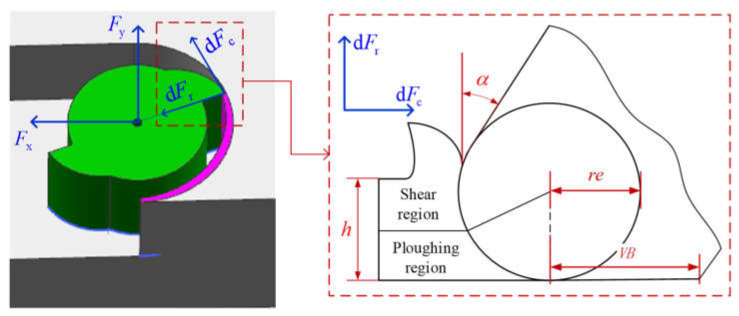
The nonlinear cutting force model in micro-milling.

By decomposing the radial and tangential forces in Equations (1) and (2) into the feed and the normal directions and integrating the elemental forces on different cutting teeth at a different depth, the theoretical forces in the feed and normal directions could be obtained. The construction of the model is shown in Figure 2. The theoretical cutting forces are as follows:

(4) $$F_{x}\left(\theta \right)=\sum_{k=1}^{M}\int_{z=0}^{d}\left[dF_{c}\cdot \cos\left(\theta_{k}^{z}\right)+dF_{r}\cdot \sin\left(\theta_{k}^{z}\right)\right]$$

(5) $$F_{y}\left(\theta \right)=\sum_{k=1}^{M}\int_{z=0}^{d}\left[dF_{c}\cdot \sin\left(\theta_{k}^{z}\right)-dF_{r}\cdot \cos\left(\theta_{k}^{z}\right)\right]$$

## 3. Tool Wear Monitoring with Nonlinear Cutting Force Model

This study focuses on the tool wear monitoring of micro-milling tools with two flutes which are commonly used in the micro-milling process. The average flank wear width of the two teeth is adopted to represent the tool wear condition of the micro-milling tool. In practice, affected by the tool runout, the tool wear condition on the different teeth is different. In this study, the tool runout is represented by the difference between the cutting radius of different teeth, as Equation (3) shows. By this representation, the asymmetry of the cutting radii caused by the actual tool runout and the tool wear are both considered. Before monitoring tool wear condition online, the model parameters are of-line estimated with measured tool wear values and cutting force. The online tool wear monitoring is carried out by jointly estimating the cutting-edge radius, tool runout, and flank wear width based on the nonlinear cutting force model.

### 3.1. Off-Line Parameters Estimation

The model parameters in Table 1 are estimated in three steps. Firstly, the parameters $\lambda_{s}$ are estimated by minimizing the gap between the theoretical force without tool wear and the measured cutting forces generated by a fresh tool. The flank wear width of a fresh tool could be set as zero, and the cutting-edge radius of a fresh tool is the initial tool edge radius. Given the initial flank wear width, initial cutting-edge radius, and cutting parameters, the theoretical cutting force without tool wear could be regarded as a function of the tool runout and the parameters $\lambda_{s}$. The genetic optimization algorithm is adopted to estimate the parameters. The optimization criterion is:

(6) $$J\left(\Delta R,\lambda_{s}\right)={\left\|F_{x}\left(\Delta R,\lambda_{s}\right)-{\bar{F}}_{x}\right\|}_{2}^{2}+{\left\|F_{y}\left(\Delta R,\lambda_{s}\right)-{\bar{F}}_{y}\right\|}_{2}^{2}$$

Notation ΔR is the difference between the two equivalent cutting radii, which is utilized to measure the tool runout. Vectors $F_{x}$ and $F_{y}$ are the theoretical forces in a short cutting pass, vectors ${\bar{F}}_{x}$ and ${\bar{F}}_{y}$ are the measured cutting forces in a short cutting pass.

Secondly, a series of friction force coefficients are estimated by minimizing the gap between the theoretical force with tool wear and the measured cutting forces generated by the worn tool. According to the model in Section 2, the theoretical cutting force could be regarded as a function of the tool runout, effective cutting-edge radius, and the friction force coefficients, with given cutting parameters and the pre-estimated parameters $\lambda_{s}$. Therefore, the optimization criterion in the second step could be written as:

(7) $$J\left(\Delta R,r_{e},K_{c,vb},K_{r,\mathrm{vb}}|\lambda_{s}\right)={\left\|F_{x}\left(\Delta R,{\bar{r}}_{e},K_{c,vb},K_{r,\mathrm{vb}}|\lambda_{s}\right)-{\bar{F}}_{x}\right\|}_{2}^{2}+{\left\|F_{y}\left(\Delta R,K_{c,vb},K_{r,\mathrm{vb}}|\lambda_{s}\right)-{\bar{F}}_{y}\right\|}_{2}^{2}$$

Finally, the parameters $\lambda_{v}$ are estimated by fitting the friction coefficient functions. The input of the friction coefficient function is the measured flank wear width, and the output is the friction force coefficient estimated in the second step. By fitting the relationship between the friction force coefficient and the flank wear width, the radial friction stress $\sigma_{v}$, the tangential friction stress $\tau_{v}$, and the width of the elastic contact region $VB^{*}$ in the parameter set $\lambda_{v}$ could be obtained. The friction coefficient functions are shown in Equations (8) and (9).

(8) $$K_{c,\mathrm{vb}}\left(VB|\lambda_{v}\right)=\{\begin{array}{cc} \frac{\tau_{v}}{3}*VB & VB<VB^{*}\\ \tau_{v}*(VB-\frac{2}{3}VB^{*}) & VB\geq VB^{*} \end{array}$$

(9) $$K_{r,\mathrm{vb}}\left(VB|\lambda_{v}\right)=\{\begin{array}{cc} \frac{\sigma_{v}}{3}*VB & VB<VB^{*}\\ \sigma_{v}*(VB-\frac{2}{3}VB^{*}) & VB\geq VB^{*} \end{array}$$

**Figure 3:**
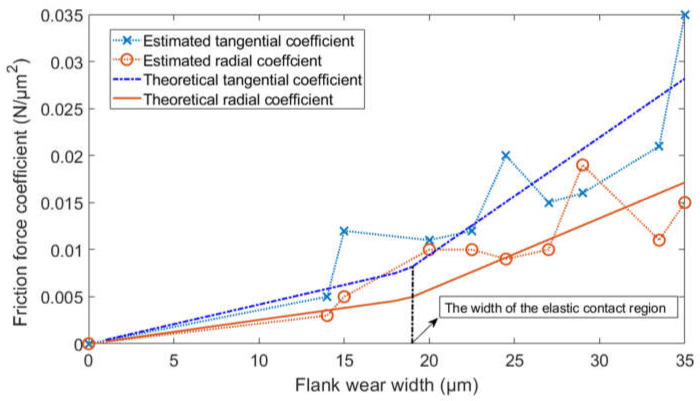
The fitting of the friction force coefficient function.

The genetic optimization algorithm is adopted to fit the friction coefficient functions. The optimization criterion is shown in Equation (10). The fitting process is shown in Figure 3.

(10) $$J\left(\lambda_{v}\right)=\sum_{i=1}^{N}\left[{\left|K_{c,\mathrm{vb}}\left(VB_{i}|\lambda_{v}\right)-K_{c,vb}^{i}\right|}^{2}+{\left|K_{r,\mathrm{vb}}\left(VB_{i}|\lambda_{v}\right)-K_{r,vb}^{i}\right|}^{2}\right]$$

Notation $VB_{i}$ is the measured flank wear width at the *i-*th cutting pass. Notations $K_{r,vb}^{i}$ and $K_{c,vb}^{i}$ are the estimated friction force coefficients at the *i-*th cutting pass. Notation *N* is the number of the cutting pass.

### 3.2. On-Line Tool Wear Monitoring

The online tool wear monitoring is carried out by minimizing the gap between the theoretical forces and the measured cutting forces. The genetic optimization algorithm is adopted to jointly estimate the average flank wear width, average cutting-edge radius, and the tool runout. The optimization criterion is as follows:

(11) $$J\left(\Delta R,{\bar{r}}_{e},\bar{\mathrm{VB}}|\lambda_{s},\lambda_{v}\right)={\left\|F_{x}\left(\Delta R,{\bar{r}}_{e},\bar{\mathrm{VB}}|\lambda_{s},\lambda_{v}\right)-{\bar{F}}_{x}\right\|}_{2}^{2}+{\left\|F_{y}\left(\Delta R,{\bar{r}}_{e},\bar{\mathrm{VB}}|\lambda_{s},\lambda_{v}\right)-{\bar{F}}_{y}\right\|}_{2}^{2}$$

By minimizing the optimization criterion in Equation (11) according to the genetic optimization algorithm, the optimum average flank wear width, average cutting-edge radius, and the tool runout could be obtained. The effectiveness of the optimization-based monitoring method is validated by comparing the estimated flank wear width and the measured flank wear width. 

## 4. Experimental Validation

### 4.1. Experimental Setup

**Figure 4:**
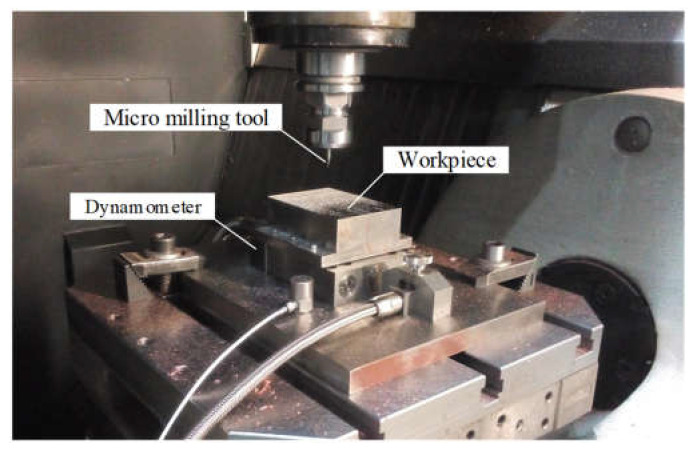
Experimental setup.

Micro slot milling experiments are conducted to validate the effectiveness of the proposed monitoring method. A total of 3 experiments are carried out under different spindle speeds, axial cutting depth, and feed per tooth. The cutting conditions of the 3 experiments are listed in Table 2. In each experiment, two micro-milling tools are used to machine the slots. The experimental data for one tool is adopted to estimate the model parameters, and the other tool is used to validate the proposed monitoring method. Ten slots are machined for each tool. A ten3-centimetre-long slot is machined at one cutting pass. The machine used in the experiments is MIKRON HSM600U vertical milling machine. The tool is C-CES-2005–0150 tool produced by UNION TOOL. The geometrical parameters of the tool are listed in Table 3. Steel AISI4340 is used as workpiece material. The cutting force in three orthogonal directions is measured with a Kistler9119AA2 3-channel dynamometer. The experimental setup is shown in Figure 4.

### 4.2. Results of Parameters Estimation and Cutting Force Prediction

The estimated parameters are listed in Table 4. Comparing the stresses in different regions, it could find that the stress in the ploughing region is much greater than the stresses in the shear region and the flank wear region. This means that the cutting force of micro-milling is most concentrated in the ploughing region. 

**Figure 5:**
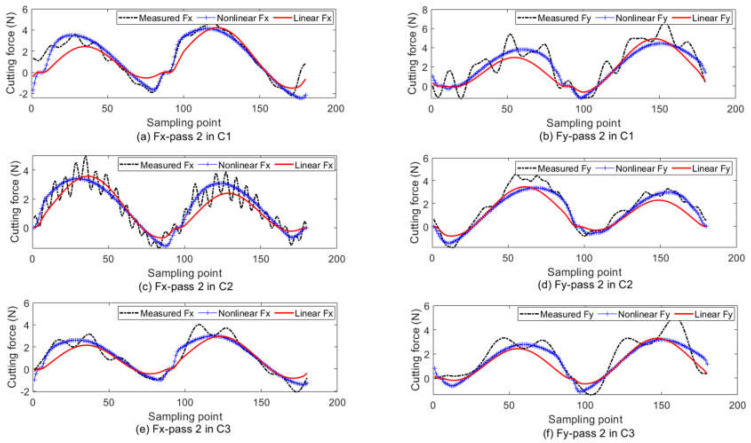
The cutting force prediction results. (**a**) F_x_ at pass 2 in C1; (**b**) F_y_ at pass 2 in C1; (**c**) F_x_ at pass 2 in C2; (**d**) F_y_ at pass 2 in C2; (**e**) F_x_ at pass 2 in C3; (**f**) F_y_ at pass 2 in C3.

The theoretical cutting force with the estimated values in Table 4 as the model parameters is shown in Figure 5. Both the proposed nonlinear force and the traditional linear force are presented. In the linear model, the cutting force in the tool-chip contact region is linear to the uncut chip thickness. The linear model with dual cutting force coefficients [14,26], which has been widely used for predicting the micro-milling force, is compared to the proposed nonlinear model. The related error defined in Equation (12) is adopted to measure the prediction accuracy. The related error is listed in Table 5. It clearly shows that the nonlinear model has a better prediction result than the linear model.

(12) $$Error=\frac{{\left\|F_{x}-{\bar{F}}_{x}\right\|}_{2}^{2}+{\left\|F_{y}-{\bar{F}}_{y}\right\|}_{2}^{2}}{{\left\|{\bar{F}}_{x}\right\|}_{2}^{2}+{\left\|{\bar{F}}_{y}\right\|}_{2}^{2}}$$

### 4.3. Tool Wear Monitoring Results

**Figure 6:**
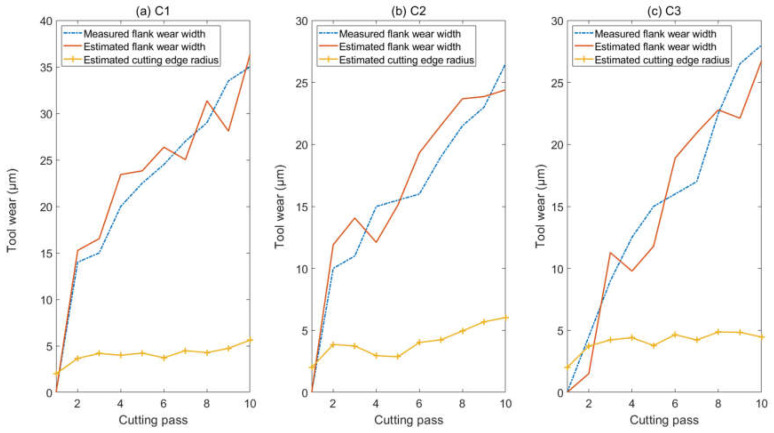
The estimated flank wear width and the cutting-edge radius. (**a**)Tool wear in C1; (**b**) Tool wear in C2; (**c**) Tool wear in C3.

The monitoring value is listed in Table 6. As the tool is fresh at the first pass, the tool wear condition at the first pass is not monitored. The flank wear width at the first pass is zero, and the cutting-edge radius at the first pass is 2 μm, as Table 3 shows. As the results show, the proposed nonlinear model is efficient to monitor the tool wear condition of micro-milling. The monitoring results are presented in Figure 6. It shows that the flank wear width has an obvious increasing tendency with the cutting time, and the effective cutting-edge radius is relatively stationary in the machining process. Because the uncut chip thickness is small, the elastic recovery part has a high portion in the chip load and has a significant effect force on the flank region. Therefore, flank wear is the main wearing form in this study. In practice, the cutting edge also wears in micro-milling. If the flank wear does not exist, the cutting-edge wear may be obvious. Due to the compensation effect of flank wear on cutting-edge wear, the cutting-edge radius does not have an obvious increasing trend. This explains why the estimated cutting-edge radius is nearly stationary.

It could be noticed that experiment C1 has the most severe flank tool wear among the three experiments. Generally, the tool wear increases with the cutting distance. Denoting the total length of the 10 slots by *L* = 30 cm, the diameter of the cutting tool by *D* = 0.5 mm, and the number of the tooth by *M* = 2, the practical cutting distance of one tooth (*CD*) could be approximately calculated by Equation (13). The practical cutting distances per tooth for the 3 experiments are listed in Table 7. As the cutting distance of C1 is longer than C3, the tool wear of C1 is more severe than C3. As the feed speed of C1 is greater than C2, the tool wear of C1 is more severe than C2. This explains why C1, which has the middle feed speed and cutting distance, has the most severe flank wear after cutting the 10 slots.

(13) $$CD=\frac{L}{M\cdot f_{z}}\times \frac{\pi D}{2}$$

The tool wear condition monitoring is also carried out by the traditional linear cutting force model with dual cutting force coefficients. The absolute error, which is defined as the absolute value of the difference between the monitored flank wear width and the measured flank wear width, is adopted to measure the monitoring accuracy. The mean absolute error is listed in Table 8. It clearly shows that the nonlinear cutting force model is more efficient to monitor the micro-milling tool wear. In regular milling, the uncut chip thickness is much greater than the cutting-edge radius, and the nonlinear effect caused by the cutting-edge radius could be neglected. Thus, adopting the linear cutting force model may have a good monitoring result in regular milling. With the decreasing of the feed per tooth, the uncut chip thickness decreases, the nonlinear effect becomes significant and the monitoring error of the linear model increases. This could be noticed in Table 8 which shows that the monitoring error of the linear model decreases with the feed per tooth. 

## 5. Conclusions

To accurately monitor the tool wear condition, a mechanistic cutting force model considering the nonlinear effect of tool wear on the micro-milling force is constructed. The tool wear monitoring is carried out according to the genetic optimization approach by which the gap between the theoretical force and the measured force is minimized. Compared to the traditional linear model, the nonlinear model has more accurate results for cutting force prediction and tool wear monitoring. Some conclusions are as follows.

(1) The force prediction accuracy and tool wear monitoring accuracy of the nonlinear model improved compared with the linear model.
(2) The flank wear width increases with the cutting time, and the effective cutting-edge radius does not have an obvious increasing trend due to the compensation effect of the flank wear on the cutting-edge wear.
(3) The nonlinear effect increases as the feed per tooth decreases, and the monitoring accuracy of the linear model increases with the feed per tooth.

The proposed nonlinear force model is efficient to estimate the average tool wear condition on the multi-tooth, but it fails to monitor the specific tool wear condition on different teeth due to averaging representation of the multi-tooth wear. In future work, the proposed monitoring approach will be extended to estimate the tool wear of multi-tooth, such that the condition of every cutting tooth is accurately monitored.

## Figures and Tables

**Table 1:** The parameters of the nonlinear cutting force model for micro-milling.

Parameter		Description	Unit
$\lambda_{s}$	$\tau_{s}$	shear stress	GPa
	β_s_	friction angle	deg
	σ_m_	ploughing coefficient	GPa
	$\tau_{m}$	friction coefficient in ploughing region	GPa
$\lambda_{v}$	σ_v_	radial friction stress	GPa
	$\tau_{v}$	tangential friction stress	GPa
	VB*	the width of the elastic contact region	μm

**Table 2:** Cutting conditions.

Cutting Condition	Spindle Speed (rpm)	Cutting Speed (m/min)	Axial Cutting Depth (μm)	Feed Speed (mm/min)	Feed per Tooth (μm/Tooth)
C1	18,000	28.27	80	144	4
C2	24,000	37.70	100	96	2
C3	30,000	47.12	60	360	6

**Table 3:** Parameters of the micro-milling tool.

Tooth Number	Tool Diameter	Rake Angle	Clearance Angle	Initial Flank Wear Width	Initial Cutting Edge Radius
2	0.5 mm	5°	7°	0 μm	2 μm

**Table 4:** Model parameters.

Cutting Condition	Estimated with Fresh Tool				Estimated with Worn Tool		
	*β_s_*	*σ_m_*	$\tau_{m}$	$\tau_{s}$	*σ_v_*	$\tau_{v}$	VB*
C1	0.56	24.98	15.03	1.01	0.76	1.25	18.68
C2	0.48	25.71	17.24	1.04	1.81	2.62	16.62
C3	0.52	27.82	23.12	1.02	2.22	2.98	18.77

**Table 5:** The error of cutting force prediction.

Cutting Pass	Nonlinear Force Model	Linear Force Model
C1	7.85%	12.25%
C2	6.11%	8.94%
C3	9.68%	12.96%

**Table 6:** Tool wear monitoring results.

Cutting Pass		Pass 2	Pass 3	Pass 4	Pass 5	Pass 6	Pass 7	Pass 8	Pass 9	Pass 10
C1	ΔR (μm)	−0.28	−0.43	0.38	−0.49	−0.36	0.32	0.55	0.34	0.34
	${\bar{r}}_{e}$(μm)	3.65	4.18	4.00	4.22	3.72	4.48	4.28	4.73	5.62
	$\bar{VB}$(μm)	15.26	16.54	23.43	23.83	26.37	25.03	31.35	28.09	36.32
	*VB*(μm)	11.92	15.00	20.00	22.50	24.50	27.00	29.00	33.50	35.00
C2	ΔR (μm)	−0.22	−0.32	0.24	0.31	0.47	0.41	0.00	0.28	0.49
	${\bar{r}}_{e}$(μm)	3.86	3.74	2.96	2.87	4.02	4.24	4.95	5.69	6.03
	$\bar{VB}$(μm)	11.90	14.07	12.10	15.10	19.32	21.53	23.68	23.85	24.40
	*VB*(μm)	10.00	11.00	15.00	15.50	16.00	19.00	21.50	23.00	26.50
C3	ΔR(μm)	−0.19	−0.40	0.53	0.63	0.64	0.65	−0.54	−0.60	−0.61
	${\bar{r}}_{e}$(μm)	3.73	4.23	4.41	3.78	4.65	4.22	4.87	4.85	4.46
	$\bar{VB}$(μm)	1.51	11.27	9.78	11.79	18.90	20.93	22.78	22.11	26.74
	*VB*(μm)	4.50	9.00	12.50	15.00	16.00	17.00	22.50	26.50	28.00

**Table 7:** The cutting distance per tooth.

Cutting Condition	Cutting Distance per Tooth
**C1**	29.45 m
**C2**	58.90 m
**C3**	19.64 m

**Table 8:** The average tool wear monitoring error.

Cutting Pass	Monitoring via Nonlinear Force Model	Monitoring via Linear Force Model
C1	2.51 μm	4.30 μm
C2	2.14 μm	4.45 μm
C4	2.66 μm	3.86 μm
